# Cancer Drug Development in Never-Smoker Lung Cancer: Targeted and Immune-Based Therapeutic Strategies

**DOI:** 10.32604/or.2026.080024

**Published:** 2026-07-16

**Authors:** Cristian Cojocaru, Marcel Costuleanu, Ovidiu Rusalim Petriș, Ruxandra Cojocaru, Decebal Vasîncu, Elena Cojocaru

**Affiliations:** Grigore T. Popa University of Medicine and Pharmacy, Iasi, Romania

**Keywords:** Never-smoker lung cancer, targeted therapy, tyrosine kinase inhibitors, immune checkpoint inhibitors, precision oncology

## Abstract

Lung cancer in individuals who have never smoked (LCINS) represents a clinically and biologically distinct subset of non–small cell lung cancer, driven predominantly by oncogenic alterations rather than tobacco-related mutagenesis. This review aims to summarize current and emerging targeted and immune-based therapeutic strategies in LCINS individuals. These patients present a molecular profile that differs substantially from tobacco-associated disease and has direct consequences for treatment selection. Evidence published over the past five years has clarified how these molecular features shape treatment response and resistance in this setting. Particular attention is given to tumors with alterations in epidermal growth factor receptor, anaplastic lymphoma kinase, c-ros oncogene 1, rearranged during transfection, Mesenchymal–Epithelial Transition (MET) exon 14 skipping mutation, human epidermal growth factor receptor 2, valine-to-glutamic acid substitution at codon 600 of the BRAF gene (BRAF V600E), and neurotrophic tyrosine receptor kinase, which together comprise the dominant driver landscape in never-smoker lung cancer. Although third-generation tyrosine kinase inhibitors have markedly improved response rates in several of these subgroups, long-term disease control is frequently compromised by acquired resistance, and heterogeneous drug exposure, particularly in the central nervous system. By contrast, immune checkpoint inhibitors have yielded limited benefit, in keeping with the low mutational burden and generally low baseline immune activation observed in most LCINS tumors. As a result, alternative approaches such as antibody–drug conjugates, bispecific antibodies, and adoptive cellular therapies are being evaluated to address gaps left by existing treatments.

## 1 Introduction

Lung cancer remains the leading cause of cancer-related mortality worldwide, accounting for more than two million deaths each year [1]. Despite the continued global decline in smoking prevalence, a growing proportion of lung cancers now occur in individuals who have never smoked (LCINS). Population-based registries estimate that 10–25% of all lung cancers arise in never-smokers, representing the fifth leading cause of cancer-related death globally [2,3]. As tobacco-associated cases decline, the absolute number of LCINS continues to increase, underscoring its significance as an emerging public health challenge. In most epidemiologic studies, never-smokers are defined as individuals who have smoked fewer than 100 cigarettes during their lifetime. In many datasets, smoking status is self-reported, which may introduce some variability in classification.

LCINS differs fundamentally from smoking-related lung cancers in its epidemiology, biology and therapeutic responsiveness. Affected patients are more frequently female, of East Asian ancestry and often younger than their smoking counterparts, with several epidemiological studies reporting a substantially higher proportion of lung cancer cases occurring in never-smokers in East Asian populations [2,4,5,6,7]. Tumors in LCINS are overwhelmingly adenocarcinomas, whereas squamous cell carcinoma and other smoking-associated histological subtypes are rarely diagnosed in this population [8]. These tumors are characterized by a genomic landscape enriched for actionable oncogenic drivers, including epidermal growth factor receptor (EGFR), anaplastic lymphoma kinase (ALK), c-ros oncogene 1 (ROS1), rearranged during transfection (RET), mesenchymal-epithelial transition factor exon 14 skipping mutation (METex14), human epidermal growth factor receptor 2 (HER2), and selected Kirsten rat sarcoma viral oncogene homologue (KRAS) variants, with a mutational spectrum distinct from that observed in smokers [9]. Large-scale genomic analyses indicate that approximately 70–75% of LCINS harbor identifiable driver alterations, compared with ~45% of smoking-related adenocarcinomas. In younger never-smokers (<40 years), the prevalence of targetable oncogenic events exceeds 80%, reinforcing the concept that LCINS is predominantly a driver-defined disease [2,9,10]. These constraints argue for a more deliberate use of immunotherapy in lung cancer in LCINS, favouring combination strategies over programmed cell death-ligand 1-PD-L1-blockade with a single agent [11]. Such features are thought to underlie the limited efficacy of immune checkpoint inhibitor (ICI) monotherapy in never-smokers, where real-world analyses report response rates of approximately 14% in never-smokers versus 36% in smokers, and shorter progression-free and overall survival [3].

In *EGFR*-mutated disease, third-generation of tyrosine kinase inhibitors (TKIs) such as osimertinib extend median progression-free survival (PFS) to 18.9 months in first-line trials [12]. Next-generation ALK inhibitors—alectinib, brigatinib, and lorlatinib—achieve 24–34 months PFS in molecularly selected populations enriched for never-smokers [13,14,15]. Similarly, selective RET inhibitors such as selpercatinib and pralsetinib, which target oncogenic fusions characteristic of never-smoker adenocarcinoma, have demonstrated objective response rates (ORR) exceeding 80% and median PFS of 16–22 months [16,17]. 

This review is focused on current and emerging therapeutic strategies in LCINS, considering the influence of molecular and immunologic features on treatment and drug development.

## 2 Methods

A narrative review of the literature was conducted using the PubMed/MEDLINE database to identify articles related to LCINS and drug development strategies. The literature search covered articles published between January 2020 and January 2025. The search included the following terms: “lung cancer in never-smokers,” “lung cancer never smoker,” “EGFR inhibitors,” “ALK inhibitors,” “targeted therapy,” “drug development,” and “precision medicine.” The search identified 211 articles. After screening titles and abstracts, 83 articles were included. The review focused on studies describing the molecular characteristics of LCINS, mechanisms of drug resistance, and clinical trials relevant to this population. Recent clinical trials, translational studies, and review articles were prioritized. Articles focusing primarily on smoking-related lung cancer were excluded. Additional relevant articles were identified by screening the reference lists of key publications.

## 3 Therapeutic Landscape and Clinical Evidence

### 3.1 Study Selection and Characteristics

The included studies evaluated targeted therapies, immunotherapies, and emerging treatment modalities for NSCLC in never-smokers. Driver alterations including EGFR, ALK, ROS1, RET, METex14–skipping, HER2, valine-to-glutamic acid substitution at codon 600 of the BRAF gene (BRAF V600E), and neurotrophic tyrosine receptor kinase (NTRK) fusions are disproportionately common in never-smokers and underpin the substantial clinical efficacy of genotype-matched TKIs [2,4,9]. In contrast, KRAS G12C is infrequently observed in this population. Baseline characteristics across these trials consistently reveal a predominance of female patients and a high prevalence of adenocarcinoma histology [5,6,7,8]. Across pivotal trials and real-world cohorts, never-smokers frequently represent a substantial proportion (often exceeding 50%) of patients receiving genotype-matched targeted therapies, primarily TKIs. For example, in the real-world FLOWER cohort of first-line osimertinib, 54.7% of patients had never smoked and only 7.9% were current smokers [18]; in the ACHIEVE trial of high-dose aumolertinib, 73% of participants were never-smokers [19], and the LIBRETTO-001 selpercatinib cohort reported that approximately 70% of patients [20]. In contrast, immunotherapy studies often enrol fewer never-smokers because high tumor mutational burden (TMB), a key contributor to checkpoint inhibitor responsiveness, is less common in this group [21]. When available, we extracted ORR, PFS, median overall survival (OS), hazard ratios (HRs), and grade ≥ 3 adverse events (AEs). Comparisons between never-smokers and smokers were performed within individual studies where such stratified data were reported.

### 3.2 Targeted Therapies

#### 3.2.1 EGFR-Mutated LCINS

Activating *EGFR* mutations represent the most prevalent oncogenic drivers in LCINS. Between 55% and 73% of patients enrolled in the FLAURA, AENEAS, and FURLONG trials were never-smokers [18,19,22]. Third-generation EGFR TKIs consistently produced high ORR and durable disease control. Aumolertinib and furmonertinib demonstrated comparable efficacy in the AENEAS and FURLONG trials, respectively, whereas osimertinib achieved an ORR of 80% and a median PFS of 18.9 months in the FLAURA trial [23,24]. HR analyses consistently suggest greater relative benefit in never-smokers compared with smokers (HR for progression 0.32 vs. 0.54) [25]. New combination strategies have recently demonstrated improved disease control: FLAURA2 showed that adding platinum–pemetrexed to osimertinib prolonged median PFS from 16.7 to 25.5 months (HR 0.62) [26], while MARIPOSA reported that amivantamab plus lazertinib increased median PFS from 16.6 to 23.7 months [27]. Notably, the MARIPOSA trial enrolled a cohort in which only 31% of participants had a history of smoking, underscoring the relevance of these findings to never-smokers. Collectively, these data position *EGFR*-mutated LCINS as a paradigm for resistance-informed, biomarker-driven drug development.

#### 3.2.2 ALK Rearrangements

ALK fusions account for approximately 5% of NSCLC and are strongly enriched in younger patients and never-smokers [28]. ALK-rearranged NSCLC is characterized by high sensitivity to TKIs, a high incidence of central nervous system (CNS) involvement, and the stepwise emergence of on-target resistance mutations that have influenced successive generations of ALK inhibitors. In the phase III CROWN trial, first-line lorlatinib decreased the risk of disease progression by 81% compared with crizotinib (HR 0.19), resulting in a 4-year PFS rate of 63% and a 5-year OS rate of 78% [15,29]. Second-generation ALK inhibitors have also shown durable clinical benefit. In the long-term follow-up of ALEX and J-ALEX, alectinib significantly extended PFS compared with crizotinib, with 5-year OS rates of approximately 60% [30].

Similarly, brigatinib demonstrated sustained systemic and intracranial disease control in randomized studies. In pivotal trials and real-world cohorts, 70–85% of patients with ALK-rearranged NSCLC are never-smokers or light smokers [31]. According to this biological profile, next-generation ALK inhibitors have achieved intracranial objective response rates exceeding 70%, reflecting improved CNS penetration and activity against resistant ALK variants. From a drug-development perspective, ALK-rearranged NSCLC is a classic example of therapeutic sequencing based on resistance. Understanding the structure of ALK resistance mutations has directly informed inhibitor design, which has led to longer disease control across many treatment lines [30]. A meta-analysis indicated that smoking status does not substantially alter the overall efficacy of ALK-TKIs; however, exploratory analyses suggested possible variations in relative performance among individual agents, with alectinib potentially preferred in smokers and lorlatinib in never-smokers [32].

#### 3.2.3 ROS1 Fusions

ROS1 rearrangements are present in approximately 1–2% of NSCLC cases and in about 2.6% of LCINS cases [33,34]. ROS1-rearranged lung cancer has been consistently included in biomarker-driven trials, reflecting its marked sensitivity to targeted inhibition. Crizotinib, ceritinib, and entrectinib were the first ROS1 TKIs evaluated in clinical studies. In treatment-naïve patients, ORR ranged from 65% to 86%, with median PFS of 15–26 months [35,36,37]. However, disease control was not sustained in all patients. Over time, progression frequently occurred, most often in the CNS, which emerged as a clinically relevant site of failure, particularly among patients with brain metastases present at diagnosis or developing during therapy [38].

With longer follow-up, the limitations of earlier ROS1 inhibitors—especially at the level of the CNS—became increasingly evident. This led to the clinical evaluation of repotrectinib, which in the TRIDENT-1 study produced ORR in 79% of treatment-naïve patients and a median PFS exceeding 35 months [39]. Notably, activity was maintained in patients with brain metastases. 

Taletrectinib was evaluated in separate clinical studies. An ORR above 90% was reported. Median PFS was not reached at the time of data cut-off and remained longer than 30 months [31]. These results were observed in populations that included patients with prior ROS1 inhibitor exposure and CNS disease. Resistance to first-generation ROS1 inhibitors has been associated with solvent-front mutations, including ROS1 G2032R. These mutations reduce sensitivity to earlier agents and represent a common mechanism of treatment failure. Newer inhibitors were designed to retain activity against resistant variants and to improve target coverage.

After progression on prior ROS1 TKIs, further targeted treatment was possible in a subset of patients. In previously treated populations, lorlatinib and repotrectinib were associated with ORR of 35–38% and median PFS of 8–9 months [31]. These findings are consistent with the feasibility of sequential ROS1-directed therapy in selected patients with LCINS.

#### 3.2.4 RET Fusions

Selective RET TKIs have substantially altered the treatment options available for patients with RET fusion–positive NSCLC. This molecular subtype is encountered infrequently in routine practice, but when present it is most often seen in adenocarcinoma and patients who are light or never smokers [40]. Before the introduction of selective RET inhibitors, therapeutic approaches relied mainly on multikinase inhibitors with partial RET activity. The administration of these pharmacological agents was limited by modest efficacy and off-target toxicity, which often prevented prolonged treatment.

Selpercatinib was the first highly selective RET inhibitor to demonstrate consistent and durable clinical activity. In the phase I/II LIBRETTO-001 trial, which enrolled 247 patients with RET fusion–positive NSCLC, approximately 70% of participants were never-smokers. In 84% of patients treated in the first line, objective responses were observed, with a median PFS of 22.0 months. Patients who had previously received systemic therapy also benefited from an ORR of 61% and a median PFS of 24.9 months [20,41]. These results support the concept that RET-rearranged tumors remain highly dependent on the pathway of action across all lines of treatment. In the phase III LIBRETTO-431 study, selpercatinib was compared to platinum-based chemotherapy with or without pembrolizumab. Treatment with selpercatinib resulted in higher ORR and a clear prolongation of PFS (24.8 vs. 11.2 months; HR 0.46, 95% CI 0.31–0.70) [42]. Nearly two-thirds of enrolled patients were never-smokers, consistent with the known distribution of RET fusions in NSCLC. The safety profile of selpercatinib has been comparable across studies. Hypertension and elevations in liver enzymes were the most frequent grade ≥ 3 AEs, while clinically relevant QTc prolongation was less common. In most cases, these events were manageable with dose adjustment or temporary interruption, allowing continued treatment in routine practice [16,40,41,42].

In this molecular subset, pralsetinib has had similar effects. In the ARROW study, 72% of patients who had never been treated before and 59% of patients who had been treated before had objective responses. The median PFS was between 13 and 16.5 months [17]. Although outcomes were not analysed according to smoking status, the enrolled population reflected the typical clinical profile of RET-rearranged NSCLC.

Overall, RET fusion–positive lung cancer provides a clear example of how selective inhibition of a single oncogenic driver can translate into meaningful and durable disease control. In never-smoker populations, where benefit from immunotherapy is often limited, RET-targeted therapies have become a practical illustration of precision oncology applied to everyday clinical care.

#### 3.2.5 METex14-Skipping

METex14-skipping occurs in approximately 2–4% of NSCLC and is typically associated with older age and heterogeneous smoking histories; adenocarcinoma predominates, and sarcomatoid features are reported at a higher frequency than in unselected NSCLC [43,44]. The percentage of patients with METex14-mutant LCINS varies across studies (36–64%) [45,46]. Although METex14 alterations are less enriched in never-smokers than EGFR or ALK rearrangements, a clinically relevant minority of cases occurs in never-smokers; therefore, METex14 should remain part of the differential when profiling LCINS lacking established drivers such as EGFR or ALK alterations [11,43]. 

In the phase II VISION study [47], patients with METex14–skipping NSCLC treated with tepotinib experienced tumor responses both in the first-line setting and after prior systemic therapy. Disease control tended to be longer when treatment was initiated upfront [47]. Responses were also observed in patients with brain metastases, which is of practical relevance given the frequency of CNS involvement in this molecular subset [43,44,47]. The safety profile was generally manageable; peripheral oedema and hypoalbuminemia were the most frequently reported AEs, while grade ≥ 3 events occurred in a minority of patients. Taken together, these findings support the clinical use of selective METex14 inhibition in NSCLC, including in never-smokers when this alteration is identified [47].

In the phase II GEOMETEX14RY mono-1 study, capmatinib achieved an ORR of 68% in treatment-naïve patients with METex14–skipping NSCLC and 41% in previously treated patients. Median PFS was 12.4 months in the first-line setting and 5.4 months after prior therapy. Clinically relevant intracranial activity was observed in patients with baseline brain metastases [48,49].

In a prospective phase II study conducted in Chinese patients with METex14–skipping NSCLC who were METex14 inhibitor–naïve, savolitinib achieved an ORR of approximately 49% and a median PFS of 6.9 months [50,51]. Responses were observed across clinically relevant subgroups, including older patients and those with brain metastases at baseline [50,51]. The toxicity profile was consistent with expectations for METex14 inhibition, with peripheral oedema and elevations in liver enzymes being the most frequently reported AEs, and was generally manageable in routine clinical practice [50,51]. In parallel, broader translational and clinical analyses of savolitinib place these results in context and discuss how combination approaches are being explored to extend disease control [50,51]. 

While METex14–skipping NSCLC spans all smoking categories, a non-trivial proportion of cases occur in never-smokers; across modern METex14 TKI trials, smoking-stratified efficacy reporting remains limited, and available data do not suggest a strong differential efficacy signal according to smoking status [43,52]. Given the limited and inconsistent efficacy of ICI monotherapy in METex14–skipping NSCLC, selective METex14 inhibition is generally preferred as first-line therapy when a METex14 alteration is identified and an approved METex14 TKI is accessible, particularly in patients with CNS involvement [47,49]. 

AEs reflected on-target MET inhibition and were dominated by peripheral oedema, hypoalbuminemia, creatinine elevations—typically reflecting transporter-mediated effects rather than true renal dysfunction—and transaminase increases; severe (grade ≥ 3) events were uncommon and typically manageable with dose modification and supportive care [47,49,50].

#### 3.2.6 KRAS G12C Mutations

The recognition of KRAS G12C as a targetable alteration has broadened treatment options in NSCLC. In patients who have never smoked, however, this mutation is encountered only infrequently, with reported prevalence generally below 3%, compared with approximately 13–14% in unselected NSCLC cohorts [53,54]. As a result, clinical experience with KRAS G12C inhibitors in this population is limited. When such tumors are identified in never-smokers, treatment decisions are therefore guided largely by data generated in smoking-associated disease, and the relevance of these findings to this subgroup remains to be fully defined.

The G12C substitution introduces a cysteine at codon 12, which allows covalent binding of small-molecule inhibitors to KRAS in its inactive, GDP-bound state. This mechanism formed the basis for the development of sotorasib (AMG 510) and adagrasib (MRTX849), both of which have demonstrated activity in previously treated KRAS G12C–mutated NSCLC [54,55]. In pivotal studies, sotorasib achieved ORR in approximately one third of patients, with median PFS just under seven months. Adagrasib produced slightly higher ORR, with a similar duration of disease control.

Importantly, these trials largely enrolled patients with a history of tobacco exposure. Never-smokers accounted for a very small fraction of participants, and outcomes for this subgroup were not reported separately. As a result, the clinical relevance of KRAS G12C inhibition in LCINS remains uncertain.

Available molecular data suggest that KRAS G12C tumors in never-smokers differ from those arising in smokers, showing fewer tobacco-related mutational features and alternative transcriptional programs [53,54]. Whether these differences translate into altered sensitivity to KRAS inhibition has not yet been addressed in dedicated clinical analyses.

Moreover, the lower TMB and unique immune microenvironment typical of never-smokers could modify the interaction between targeted inhibition and immune surveillance mechanisms. Experimental studies indicate that KRAS inhibition may influence antitumor immune signalling, including effects on antigen presentation and cytokine pathways [56]. To date, however, these observations have not been confirmed in clinical series of KRAS G12C positive lung cancer from never-smokers.

ICIs are widely used in NSCLC, but their role in KRAS G12C–mutated disease among never-smokers is less clearly defined. In broader NSCLC cohorts, tumors harbouring KRAS mutations show variable sensitivity to programmed cell death protein-1 (PD-1) or programmed death-ligand 1 (PD-L1) blockade, with better outcomes reported in the presence of TP53 co-mutations and higher PD-L1 expression. In contrast, alterations in serine/threonine kinase 11 or kelch-like ECH-associated protein 1, which can also occur in patients without a smoking history, have been associated with reduced benefit from immunotherapy [57]. 

LCINS are typically characterized by low TMB, a feature that has been associated with modest activity of ICI monotherapy in KRAS G12C–mutated tumors. At present, the clinical impact of KRAS G12C inhibition in never-smokers remains modest, reflecting both the rarity of this alteration and the incomplete understanding of its immune context [58].

When KRAS mutations are identified in LCINS, they are less frequently of the G12C subtype, which is strongly associated with tobacco exposure, and may instead involve alternative codon 12 or 13 variants that are not directly targetable with current covalent inhibitors. This mutational pattern further limits the clinical applicability of KRAS-directed strategies in this setting [59].

#### 3.2.7 Other Oncogenic Drivers (HER2, BRAF V600E, NTRK)

Beyond the major driver alterations, a smaller but clinically relevant subset of LCINS is characterized by HER2 alterations (3.9%), BRAF V600E mutations (0.4%), or NTRK fusions [34,60].

HER2-driven NSCLC, most commonly through exon 20 insertions, is overrepresented in adenocarcinomas arising in never-smokers. In this setting, the antibody–drug conjugate (ADC) trastuzumab deruxtecan has demonstrated robust antitumor activity and clinically relevant PFS in the DESTINY-Lung01 trial, findings that have led to its incorporation into current treatment algorithms for HER2-positive disease [61].

BRAF V600E mutations define a smaller molecular subset of NSCLC and are encountered more often in patients without substantial tobacco exposure. Data from a multicenter Chinese real-world cohort indicate that these tumors display distinct clinical features and derive benefit from combined BRAF and mitogen-activated protein kinase (MEK) inhibition, although the proportion of never-smokers remains lower than in other oncogene-addicted subgroups [62].

NTRK fusions are rare in NSCLC, without a clear association with smoking status. When present, however, they define a subset of tumors that can respond remarkably well to tyrosine receptor kinase (TRK) inhibition. Clinical experience with larotrectinib and entrectinib shows that substantial and often prolonged disease control can be achieved, even across different tumor histologies and lines of therapy. Although NTRK-rearranged tumors represent a small proportion of LCINS, their identification is clinically meaningful given the depth of response observed with targeted therapy [2,4,9,63].

Table 1 summarizes selected trials of targeted agents used in LCINS, focusing on outcomes that are most relevant for daily clinical decision-making. Most studies derive from biomarker-selected NSCLC populations enriched for never-smokers rather than trials specifically conducted in never-smoker cohorts.

**Table 1:** Selected targeted therapy trials relevant to LCINS.

Driver	Representative Trials	Key Population/Smoking Features	Efficacy	Key Toxicities
EGFR	FLAURA—osimertinib vs. gefitinib/erlotinib [12]; FLOWER—real-world osimertinib [18]; FURLONG—furmonertinib vs. gefitinib [22]; ACHIEVE—aumolertinib [19]; befotertinib studies [23,24]; review on smoking impact [25]; FLAURA2 trial [26]	Predominantly never or light-smokers (55–73%); predominantly adenocarcinoma [2,4,5,9,18,22,25]	ORR 70–80%; median PFS 18–22 months (first-line, TKI-naïve); improved CNS activity reported; combination regimens may extend median PFS beyond 24 months [12,18,19,22,26]	Diarrhea, rash, hepatic enzyme elevations; ILD (rare) [12,18,19,22,24]
ALK	ALEX—alectinib [13]; ALTA-1—brigatinib [14]; CROWN—lorlatinib [15,29]; long-term and real-world analyses [28,30,31]; network METex14-analysis [32]	Strong enrichment in never or light-smokers (70–85%); younger age; frequent CNS involvement [2,5,28,30,31,32]	ORR 70–90%; median PFS 25–34 months (first-line); 5-year OS 60–78% [13,15,28,29,30]	Dyslipidemia and neurocognitive effects (lorlatinib); myalgia and hepatic AEs (alectinib/brigatinib) [13,15,29,30]
ROS1	Clinical trials and reviews including crizotinib, entrectinib, lorlatinib, repotrectinib, taletrectinib [33,35]	Never-smoker–predominant adenocarcinoma [2,4,9,33,35]	ORR 67–72%, median PFS 15–19 months (first-line); next-generation ROS1 inhibitors demonstrate ORR 70–90%, median PFS > 30 months in TKI-naïve disease [33,35]	Dizziness, peripheral neuropathy, hyperlipidaemia [33,35]
RET	LIBRETTO-001—selpercatinib [16,20,64]; LIBRETTO-431—selpercatinib vs. chemotherapy ± pembrolizumab [42]; ARROW—pralsetinib [17]; FDA prescribing information [65]	Predominantly never-smokers (65–70%); adenocarcinoma; often younger patients [2,4,16,17,20,42]	ORR 84%; median PFS 22–25 months (treatment-naïve); superior outcomes compared with chemotherapy [16,17,20,42,64]	Hypertension, ALT/AST elevations, QT prolongation [16,20,42,64,65]
METex14	VISION—tepotinib [47]; GEOMETRY mono-1—capmatinib [48,49]; savolitinib—phase II [50,51]; epidemiologic and real-world analyses [43,44,52]	Heterogeneous smoking histories; recurrent but non-dominant alteration in never-smokers [2,9,11,43,44,52]	ORR 46–72%; median PFS 9–16 months (treatment-naïve and previously treated populations); intracranial activity reported [47,48,49,50]	Peripheral oedema, hypoalbuminemia, creatinine elevation, transaminase increases [47,48,49,50,51]
KRAS G12C	CodeBreaK 100—sotorasib [54]; KRYSTAL-1—adagrasib [55]; biologic and mechanistic studies [53,56,57]	Predominantly smokers; <3% KRAS-mutated NSCLC in never-smokers [2,4,9,53,54,56]	ORR 37–43%; median PFS ~6–7 months in (previously treated disease) [54,55]; data largely derived from smoking-related cohorts	Diarrhea, nausea, hepatotoxicity [54,55]
HER2/BRAF/NTRK	DESTINY-Lung01—trastuzumab deruxtecan [61]; BRAF V600E real-world cohort [62]; NTRK fusions review [63]	Enriched in never-smoker adenocarcinoma; low absolute prevalence [2,4,9,61,62,63]	High ORRs and clinically meaningful PFS reported with HER-2- and NTRK-targeted therapies (predominantly in previously treated populations); established activity of BRAF/MEK inhibition in BRAF V600E NSCLC [61,62,63]	ADC-related AEs (including ILD, cytopenias); BRAF/MEK toxicities (pyrexia, rash) [61,62]

Legend. LCINS, Lung Cancer in Never-Smokers; EGFR, Epidermal Growth Factor Receptor; vs., versus; ORR, Objective Response Rates; PFS, Progression-Free Survival; TKIs, Tyrosine Kinase Inhibitors; CNS, Central Nervous System; ILD, Interstitial Lung Disease; ALK, Anaplastic Lymphoma Kinase; OS, Overall Survival; AEs, Adverse Events; ROS1, c-Ros Oncogene 1; RET, Rearranged during Transfection; ALT, Alanine Aminotransferase; AST, Aspartate Aminotransferase; QT, QT interval; FDA, Food and Drug Administration; METex14, Mesenchymal-Epithelial Transition factor exon 14 skipping mutation; NSCLC, Non–Small Cell Lung Cancer; HER2, Human Epidermal Growth Factor Receptor 2; NTRK, Neurotrophic Tyrosine Receptor Kinase; MEK, Mitogen-Activated Protein Kinase; ADC, Antibody–Drug Conjugate.

#### 3.2.8 Limited Efficacy of Immune Checkpoint Inhibitors in LCINS

ICIs have become a cornerstone of therapy for advanced NSCLC; however, their clinical benefit appears attenuated in patients who have never smoked. Across randomized trials and real-world series, never-smokers consistently derive lower ORR and shorter PFS and overall survival from PD-1/PD-L1 blockade compared with patients with a smoking history. These differences persist even when ICIs are administered in the first-line setting and cannot be fully explained by performance status or treatment line alone [66].

Several biological features characteristic of LCINS provide a plausible explanation for this reduced efficacy [3,11,66]. Compared with smoking-related lung cancer, tumors arising in never-smokers typically exhibit a lower overall mutational burden [3,66]. A low TMB level is unlikely to fully explain the limited activity of ICIs in LCINS [11,66]. Oncogenic signaling and characteristics of the tumor microenvironment may also influence sensitivity to immunotherapy [11,66]. At the tissue level, many never-smoker tumors show little evidence of an active antitumor immune response, with sparse infiltration by effector T cells and a relative predominance of regulatory immune populations [11,66]. Smoking status also appears to influence systemic immune profiles [66]. Data from studies in healthy populations indicate that active smoking is associated with measurable shifts in circulating T-cell subsets, whereas individuals who have never smoked more often maintain stable CD4^+^ and CD8^+^ T-cell proportions [67]. Together, these features are not typically associated with durable benefit from ICI monotherapy.

Importantly, the limited activity of ICIs in LCINS appears to extend beyond simple biomarker thresholds. Although PD-L1 expression can be variable and occasionally high in never-smokers, PD-L1 positivity alone has not reliably translated into robust clinical benefit in this population. This framework places molecular stratification at the center of treatment selection. In clinical studies, lung cancers driven by alterations such as EGFR, ALK, ROS1, RET, METex14–skipping, or HER2 have generally shown limited responses to ICI monotherapy. This pattern has been observed even in cases with detectable PD-L1 expression, indicating that tumor behavior in these settings is more strongly influenced by driver-dependent signaling than by endogenous antitumor immune activity [68].

These biological features have practical consequences in routine care. In LCINS, broad molecular testing should be completed before treatment decisions are finalized, as the identification of an actionable alteration supports the use of targeted therapy ahead of single-agent immunotherapy, in line with current guideline-based practice. When ICIs are introduced, this is most often in combination approaches or after exhaustion of targeted options, with an understanding that the magnitude of benefit is typically lower than that seen in smoking-related disease.

### 3.3 Emerging Modalities for Non-Smokers

In clinical practice, LCINS frequently presents a therapeutic paradox: tumors are highly dependent on specific oncogenic pathways, yet derive limited benefit from ICI. These limitations have encouraged the development of newer treatment modalities, including bispecific antibodies (bsAbs), ADC, and adoptive cellular therapies (ACT), which aim to bypass resistance to kinase inhibition or to re-engage antitumor immunity in tumors that are otherwise immunologically silent.

#### 3.3.1 bsAbs

The most clinically advanced bsAbs for NSCLC is amivantamab, a fully human IgG1 antibody targeting EGFR and MET receptors. These two signaling axes frequently cooperate in resistance mechanisms within EGFR-mutated tumors, which are strongly enriched among never-smokers [69]. By binding the extracellular domains of both EGFR and METex14, amivantamab blocks ligand activation, promotes receptor internalization and degradation, and elicits antibody-dependent cellular cytotoxicity, thereby extending efficacy beyond kinase inhibition alone.

In the pivotal CHRYSALIS trial, which included a predominantly never-smoker population with *EGFR exon 20 insertion (ex20ins)* mutations, amivantamab produced an ORR of 40%, median duration of response (DOR) 11.1 months, and PFS of 8.3 months after platinum therapy [70]. The vast majority of participants had minimal or no smoking history, reflecting the strong association of ex20ins with never-smoker biology. These results led to the first regulatory approval of a bsAbs in NSCLC, specifically targeting a genotype that occurs almost exclusively in never-smokers.

Building on these data, the PAPILLON phase III trial established amivantamab plus carboplatin/pemetrexed as a first-line standard for *EGFR* *ex20ins* NSCLC, achieving a significant median PFS benefit (11.4 vs. 6.7 months) and ORR 73% versus chemotherapy alone [71]. Because ex20ins mutations are detected overwhelmingly in never-smokers, this regimen effectively represents a combined targeted and immune-engaging strategy tailored to this population.

The integration of amivantamab with third-generation EGFR TKIs further enhances its relevance for never-smokers. The MARIPOSA and MARIPOSA-2 studies demonstrated that amivantamab plus lazertinib outperformed osimertinib monotherapy both in treatment-naïve and in post-osimertinib resistant EGFR-mutated NSCLC [72]. Most patients enrolled were never-smokers with classical *EGFR* *exon* *19* *deletion* or *L858R* mutations, confirming the therapeutic impact of bsAbs in the molecularly defined subset that typifies LCINS.

Due to the limited durability of PD-1/PD-L1 monotherapy in this biological context, there are alternative strategies for stimulating the immune system, such as bsAbs. In the multicenter phase Ib/II AK104-202 trial, cadonilimab (a PD-1/CTLA-4 bispecific antibody) achieved an ORR of 18.8%, median PFS of 4.1 months, and DOR of 6.3 months in previously treated advanced NSCLC. The median OS had not yet been reached at data cut-off (lower 95% CI 8.7 months), and immune-related AEs were consistent with combined checkpoint blockade but generally manageable [73]. Although the study population was mixed, correlative analyses showed that responses occurred mainly in tumors lacking smoking-related signatures, suggesting preferential activity in tumors without dominant smoking-related molecular signatures. By co-blocking two immune inhibitory axes within a single molecule, such bsAbs may restore immune visibility in tumors where conventional checkpoint blockade has limited effect.

From a mechanistic perspective, bsAbs address two central challenges of LCINS: pathway redundancy and immune inertia characteristic of low–TMB disease. The antitumor effects of EGFR/MET bsAbs are thought to involve suppression of bypass signaling pathways associated with TKI resistance. In contrast to smoking-related tumors, LCINS often retains intact antigen-presentation pathways and lack extensive background mutagenesis. Under these conditions, immune activation may be achievable once an effective trigger is introduced. Preclinical work suggests that amivantamab can contribute to such activation by enhancing antigen presentation and interacting favorably with PD-1 blockade, which has motivated current combination trials in EGFR-mutated NSCLC [74,75].

Agents such as zenocutuzumab (HER2/HER3) and checkpoint-directed constructs like MGD013 (PD-1/LAG-3) and AK112 (PD-1/T-cell immunoreceptor with Immunoglobulin and Immunoreceptor Tyrosine-based Inhibition Motif domains (TIGIT)) are being developed to address the molecular and immune features typical of never-smoker adenocarcinomas. HER3-directed antibodies are particularly relevant in *EGFR*- or *HER2*-altered tumors, which predominate among never-smokers, while dual-checkpoint bsAbs seek to amplify T-cell activation within low–TMB microenvironments. Early clinical data indicate acceptable safety profiles in NSCLC, with preliminary signals of antitumor activity [75,76].

#### 3.3.2 Antibody–Drug Conjugates

ADCs have become an increasingly relevant therapeutic option for patients with oncogene-driven NSCLC who experience disease progression after TKI therapy, a scenario frequently encountered in never-smokers. Tumors in this setting are often characterized by EGFR or HER3 alterations and, in some cases, METex14 amplification. The consistent expression of targetable surface antigens, together with a generally low TMB, provides a biological rationale for the use of ADC-based strategies in this population.

Among the earliest ADCs evaluated in NSCLC was the trophoblast cell surface antigen 2 (TROP2)-directed compound sacituzumab govitecan. Its activity was explored in a phase I/II study that enrolled heavily pretreated patients with metastatic disease, of whom 47 were evaluable for response. Treatment resulted in objective tumor shrinkage in 19% of patients, while disease stabilization contributed to a clinical benefit rate of 43%. Median PFS and OS were 5.2 and 9.5 months, respectively [77,78]. Although smoking status was not examined as a dedicated subgroup, the predominance of adenocarcinoma in the study population lends biological relevance to lung cancer cohorts enriched in never-smokers.

HER3-targeted delivery has attracted particular interest in EGFR-mutated disease, where receptor expression is frequent and often preserved after TKI resistance. In the HERTHENA-Lung01 study, patients with EGFR-mutated NSCLC who had previously received TKIs showed measurable antitumor activity with patritumab deruxtecan, including a confirmed ORR of 29.8%. Median PFS was 5.5 months, and median OS reached 11.9 months [79,80]. Clinical activity was documented across heterogeneous resistance settings, including tumors progressing on multiple prior TKIs. In EGFR-mutated lung cancer, a molecular subtype enriched in never-smokers, this supports HER3-directed ADCs as a post-TKI therapeutic option.

#### 3.3.3 Adoptive Cell Therapy (ACT)

ACT is being explored as a strategy to overcome primary and acquired resistance to immunotherapy in NSCLC [81]. From a biological perspective, ACT is particularly appealing in LCINS, where tumors typically lack spontaneous immune activation, with limited baseline immunogenicity [66,81]. These characteristics tend to limit endogenous immune activation, yet they do not exclude the presence of lymphocyte populations capable of recognizing tumor antigens once removed from the suppressive tumor milieu and expanded *ex vivo* [3]. Evidence for this comes from translational studies in NSCLC showing that tumor-infiltrating lymphocytes can be reliably isolated and expanded from metastatic lesions. In an analysis of 27 metastatic NSCLC samples, viable tumor-infiltrating lymphocyte (TIL) products were generated from every specimen, and antitumor reactivity—often accompanied by polyfunctional cytokine release—was observed in the majority of cases. Importantly, successful expansion was not restricted by metastatic site or prior systemic treatment exposure [81]. 

Clinical application of ACT in NSCLC is still at an early stage, but initial studies have established biological feasibility and clinical signal rather than definitive efficacy benchmarks.

In a phase I trial combining autologous TIL therapy with nivolumab in PD-1–resistant metastatic NSCLC, Creelan et al. reported confirmed responses in 3 of 13 evaluable patients (23%), including two durable complete responses, alongside tumor burden reductions in the majority of treated cases; toxicity was primarily related to lymphodepletion and interleukin-2 support [82]. More recently, a phase II multicenter study evaluating lifileucel (LN-145) monotherapy in immunotherapy-refractory metastatic NSCLC reported an ORR of 21.4%, with responses observed even in PD-L1–negative and low–TMB tumors [83].

Table 2 outlines representative clinical studies of immunotherapy and emerging treatment modalities in lung cancer among never-smokers. The included studies comprise both trials enriched for never-smokers due to oncogenic drivers and studies conducted in broader NSCLC populations.

**Table 2:** Selected immunotherapy and emerging modality studies relevant to LCINS.

Therapy/Modality	Representative Trials	Key Population/Smoking Features	Efficacy	Key Toxicities
Immune Checkpoint Inhibitors	PD-1 blockade activity in never, light, and heavy smokers [21]; translational review on immunologic challenges [3]	Never-smokers: low TMB; variable PD-L1 expression; poor ICI monotherapy response (14% ORR vs. 36% in smokers) [3,21]	Monotherapy ORR 14–20%; limited OS benefit, particularly in oncogene-driven disease; modest PFS improvement with combinations [3,21,25]	Fatigue, pneumonitis, colitis, hepatitis [3,21]
Bispecific Antibodies	CHRYSALIS—amivantamab (EGFR/MET) in EGFR ex20ins NSCLC [70]; PAPILLON—amivantamab + chemotherapy [71]; MARIPOSA—amivantamab + lazertinib [72]; PD-1/CTLA-4 bsAbs cadonilimab (AK104-202) [73]; expert reviews [74,75,76]	Predominantly never-smokers (EGFR ex20ins); low TMB and PD-L1 expresssion; immunologically ‘cold’ tumors with dominant oncogene dependence [2,3,70,71,72]	Amivantamab: ORR 40–73%, PFS 8–11 months; cadonilimab: ORR 19%, PFS 4 months; promising synergy with EGFR TKIs [70,71,72,73,75,76]	Infusion reactions, rash, paronychia (EGFR/MET bsAbs); immune-related AEs (PD-1/CTLA-4 bsAbs) [70,71,72,73]
Antibody–Drug Conjugates	Sacituzumab govitecan (TROP2 ADC) [77,78]; Patritumab deruxtecan (HER3-DXd; HERTHENA-Lung01) [79,80]; review on ADCs in lung cancer [43,52]	EGFR- or HER3-mutated tumors; strong biological relevance for never-smokers [2,4,9,77,78,79,80]	ORR 20–30% in post-TKI disease; median PFS 5–6 months; HER3-deruxtecanOS ~12 months [79,80]	Neutropenia, fatigue, nausea, ILD (rare) [77,78,79,80]
Adoptive Cell Therapy	Generation of TILs [81]; Phase I TIL + nivolumab in PD-1-resistant NSCLC [82]; Lifileucel phase II monotherapy [83]; review of ACT in lung cancer [51]	Low-TMB NSCLC (never-smoker–enriched); biologic relevance for LCINS and never-smokers [3,81,82,83]	ORR 21–23% in immunotherapy-refractory disease; durable CRs in selected cases; feasibility across tumor sites [82,83]	Lymphodepletion-and cytokine-related toxicity; febrile events; manageable with supportive care [81,82,83]

Legend. LCINS, Lung Cancer in Never-Smokers; TMB, Tumor Mutational Burden; PD-L1, Programmed Death-Ligand 1; ICI, Immune Checkpoint Inhibitor; vs., versus; ORR, Objective Response Rates; OS, Overall Survival; PFS, Progression-Free Survival; EGFR, Epidermal Growth Factor Receptor; METex14, Mesenchymal-Epithelial Transition factor exon 14 skipping mutation; ex20ins, *exon* *20* *insertion*; NSCLC, Non–Small Cell Lung Cancer; PD-1, Programmed Cell Death Protein-1; CTLA-4, Cytotoxic T-Lymphocyte-Associated Protein 4; bsAbs, Bispecific Antibodies; TKIs, Tyrosine Kinase Inhibitors; AEs, Adverse Events; TROP2, Trophoblast cell surface antigen 2; ADC, Antibody–Drug Conjugate; HER3, Human Epidermal Growth Factor Receptor 3; ILD, Interstitial Lung Disease; TILs, Tumor-Infiltrating Lymphocytes; ACT, Adoptive Cell Therapy; CRs, Complete Responses.

Studies summarized in the Table 2 include both trials enriched for never-smokers due to oncogenic drivers and trials conducted in broader NSCLC populations in which smoking status was not always analyzed separately.

**Figure 1:**
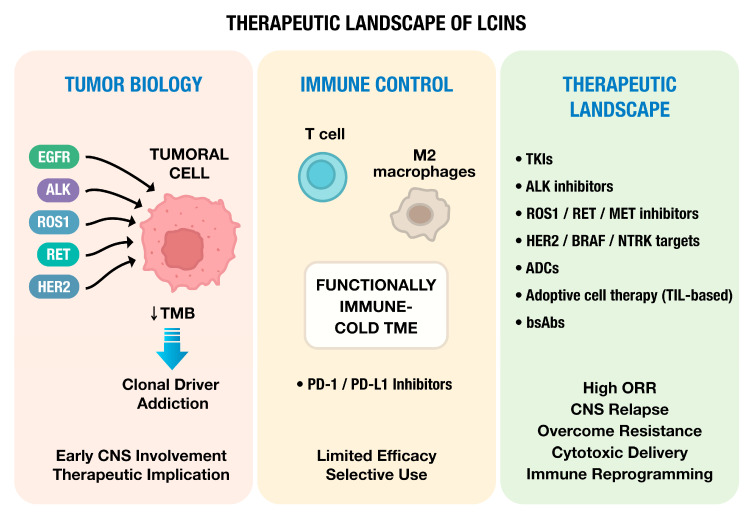
Therapeutic landscape of lung cancer in never-smokers. Legend: LCINS, Lung Cancer in Never-Smokers; EGFR, Epidermal Growth Factor Receptor; ALK, Anaplastic Lymphoma Kinase; ROS1, c-Ros Oncogene 1; RET, Rearranged during Transfection; HER2, Human Epidermal Growth Factor Receptor 2; ↓, decrease; TMB, Tumor Mutational Burden; CNS, Central Nervous System; T cell, T Lymphocytes; M2 macrophages, a subset of tumor-associated macrophages; TME, Tumor Microenvironment; PD-1, Programmed Cell Death Protein 1; PD-L1, Programmed Death-Ligand 1; TKIs, Tyrosine Kinase Inhibitors; MET, Mesenchymal-Epithelial Transition factor; NTRK, Neurotrophic Tyrosine Receptor Kinase; ADC, Antibody–Drug Conjugate; bsAbs, Bispecific Antibodies; ORR, Objective Response Rates.

Fig. 1 provides an overview of the evolving therapeutic landscape in LCINS, integrating both established and emerging treatment strategies.

## 4 Challenges and Future Prospects

Therapeutic sequencing is therefore anchored in genomic profiling [2,4,5,9,11]. Actionable alterations such as *EGFR*, *ALK*, *ROS1*, *RET*, *METex14–skipping*, *HER2*, *BRAF V600E*, and *NTRK* fusions occur in up to 70–80% of cases, supporting a paradigm shift toward genotype-guided therapy [4,5,11,33,63]. In never-smokers, third-generation EGFR TKIs such as osimertinib, aumolertinib, and befotertinib routinely produce high ORR, often exceeding 70%, with median PFS close to 20 months [12,18,19,22,24]. A comparable degree of disease control has been observed with modern ALK inhibitors, including alectinib, brigatinib, and lorlatinib, for which median PFS frequently extends beyond two years, particularly in never-smoker populations [13,14,15,28,29,30,31,32]. 

Comparable efficacy is seen with ROS1, RET, and METex14 inhibitors, where ORR frequently surpass 60–80%, with durable responses that appear particularly pronounced in this population [16,17,18,19,20,33,35,42,64]. However, despite these advances, acquired resistance and CNS progression remain major therapeutic barriers [13,15,28,29,43]. Future research must therefore focus on developing fourth-generation TKIs, dual-target combinations, and strategies that prevent or delay molecular resistance while maintaining tolerability [9,25,26]. The major resistance mechanisms and therapeutic sequencing considerations for key oncogenic drivers in LCINS are summarized in Table 3.

**Table 3:** Key resistance mechanisms and therapeutic sequencing in major oncogenic drivers in LCINS.

Driver Alteration	Common Resistance Mechanisms	CNS Considerations	Typical Therapeutic Sequencing	Drug Development Implication
EGFR mutations	On-target mutations (e.g., T790M, C797S); bypass signaling such as MET amplification [9,25,26]	CNS metastases are frequent; agents with CNS activity are preferred [12,18,26]	First-line osimertinib; subsequent therapy guided by resistance mechanisms (e.g., MET-directed therapy, chemotherapy) [12,18,26]	Development of fourth-generation EGFR inhibitors and combination strategies targeting MET [9,25,26]
ALK rearrangements	Secondary ALK kinase mutations; activation of bypass pathways [28,30]	High CNS activity is important due to frequent brain metastases [13,15,28]	First-line next-generation ALK inhibitors (e.g., alectinib or lorlatinib), with subsequent therapy guided by resistance mechanisms [13,14,29]	Sequential development of next-generation ALK inhibitors with improved CNS penetration [15,28,30]
ROS1 rearrangements	Kinase domain mutations; bypass signaling pathways [33,35,38]	CNS penetration increasingly relevant [38,39]	ROS1 inhibitors followed by next-generation agents depending on resistance patterns [33,35,39]	Development of ROS1 inhibitors active against solvent-front mutations (e.g., G2032R) [33,35,39]
MET exon 14 skipping	Secondary MET mutations; activation of alternative signaling pathways [43,47]	CNS activity under investigation [47,49]	MET inhibitors as first-line targeted therapy; subsequent treatment based on resistance or systemic progression [47,48,49]	Optimization of selective MET inhibitors and combination strategies [43,47,49]

Legend. LCINS, Lung Cancer in Never-Smokers; CNS, central nervous system; EGFR, Epidermal Growth Factor Receptor; MET, Mesenchymal-Epithelial Transition; ALK, Anaplastic Lymphoma Kinase; ROS1, c-Ros Oncogene 1; METex14, Mesenchymal-Epithelial Transition factor exon 14 skipping mutation.

It should be noted that much of the available clinical evidence discussed in this review derives from biomarker-selected NSCLC trials, in which never-smokers represent a substantial proportion of participants but are rarely analyzed as a distinct efficacy subgroup. These biological features translate clinically into reduced sensitivity to immune checkpoint blockade in never-smokers, even in the presence of PD-L1 expression [3,9,11]. The low efficacy of ICI monotherapy may be especially true among oncogene-addicted LCINS, where tumors harboring alterations in EGFR, ALK, ROS1, RET, or METex14 are likely to derive little benefit from PD-1/PD-L1 inhibitor monotherapy. Conversely, oncogene-negative tumors among never-smokers may still possess some level of immunotherapy sensitivity, especially when ICI is used in combination with chemotherapy or anti-angiogenic agents. However, the interpretation of such findings is difficult given the lack of clinical trial data on the outcome of never-smoker patients. These constraints argue for a more deliberate use of immunotherapy in LCINS, favoring combination strategies over PD-(L)1 blockade with a single agent. Approaches that integrate ICIs with anti-angiogenic agents or selected targeted therapies may help modify the tumor microenvironment and improve immune engagement in this setting [3,9,57]. A clearer understanding of how oncogenic signaling intersects with immune escape mechanisms will be essential to identify biomarkers with greater clinical relevance than PD-L1 expression or TMB alone. In this context, periods of molecular adaptation or resistance to targeted therapy—when tumor antigenicity may change—could offer a window of opportunity for enhancing sensitivity to ICI [57,73]. 

In LCINS, treatment development has increasingly moved beyond exclusive reliance on kinase inhibition. Alongside TKIs, several biologic approaches are now under clinical evaluation, most notably bsAbs, ADC, and ACT.

One of the earliest biologic strategies to show clinical activity in LCINS has been amivantamab, a bsAbs directed against EGFR and METex14. In patients with EGFR ex20ins–mutated disease, amivantamab produced meaningful tumor responses in settings where conventional TKIs have historically shown limited efficacy [63,64,65,67].

These data support the role of antibody-based targeting in molecular contexts that are less amenable to small-molecule inhibition. BsAbs targeting ICI have entered clinical testing in advanced NSCLC. Cadonilimab, a PD-1/CTLA-4 bispecific construct, has shown antitumor activity in previously treated patients, providing clinical proof that dual checkpoint inhibition can be delivered within a single agent [66,69].

ADC have likewise been evaluated in heavily pretreated NSCLC, expanding systemic treatment options beyond kinase inhibitors. Trastuzumab deruxtecan, sacituzumab govitecan, and patritumab deruxtecan have shown ORR in tumors expressing HER2, TROP2, or HER3, respectively, including molecular subtypes that are more frequently observed in never-smokers [54,70,71,72,73]. These agents provide an alternative means of drug delivery in tumors that remain dependent on surface antigen expression after failure of kinase inhibitors.

As their use increases, attention has shifted toward toxicity management—particularly interstitial lung disease—and toward identifying biomarkers that better predict ADC sensitivity [39,54].

In parallel, ACT is re-emerging as a potential option for selected patients with LCINS. Early clinical studies of tumor-infiltrating lymphocyte therapy have reported durable responses in PD-1–refractory NSCLC, demonstrating that effective TIL products can be generated even from limited biopsy material [74,75,76]. Integration of ACT with targeted and antibody-based strategies may represent an additional therapeutic avenue for selected patients with LCINS, provided that manufacturing complexity and patient selection barriers are addressed [45,75,76].

Current evidence indicates that resistance-informed trial designs and biomarker-guided combinations will be required to further improve outcomes.

These observations highlight several priorities for future research in LCINS. In LCINS, treatment decisions are frequently guided by molecular findings, reflecting the high rate of actionable alterations in this population. Thus, comprehensive molecular profiling, preferably using next-generation sequencing panel testing, is essential for the accurate diagnosis of LCINS and for identifying actionable genomic alterations that may guide targeted therapy and the development of new therapeutic strategies. Broad next-generation sequencing approaches have therefore become particularly useful, as they allow detection of less common events such as gene fusions, exon insertions, or exon-skipping variants that may not be captured by focused assays [4,11,43]. These considerations also illustrate the impact of the biological features of LCINS on current drug development strategies. First, the high rate of actionable driver mutations supports the design of biomarker-driven clinical trials. The development of resistance mechanisms during the course of targeted therapies has also informed the design of next-generation inhibitors. Finally, the high rate of brain metastases in oncogene-driven cancer has informed the design of inhibitors with activity in the central nervous system. As a result, clinical management in never-smokers has largely shifted toward early use of matched targeted therapies, whereas ICIs are more often introduced after targeted options or in selected cases with supportive biomarkers [3,21]. Advances in this area have relied on parallel progress in genomic and translational research, which has clarified patterns of resistance and immune escape in oncogene-driven lung cancer [9,11,57]. Serial sampling and integrative molecular analyses in clinical studies have contributed to a better understanding of clonal evolution during treatment. More recently, exploratory computational approaches have been applied to these datasets with the aim of improving prediction of resistance and treatment response at the individual patient level [9,23].

This review is subject to variability in study design and reporting within the available literature. Most evidence is derived from heterogeneous clinical trials and retrospective series, and smoking status is not consistently reported or analyzed as an independent variable. As a result, several observations relevant to LCINS rely on indirect comparisons rather than smoking-specific endpoints. Even with these constraints, the accumulated data support a consistent biological pattern, in LCINS is dominated by oncogenic driver alterations and displays limited intrinsic immunogenicity, setting it apart from tobacco-associated NSCLC [2,4,9]. Future research must address several critical gaps, including the development of fourth-generation TKIs, rational TKI-ADC or TKI-ICI combinations to overcome resistance, and prospective trials stratified by smoking status to validate efficacy in true never-smoker populations [9,25,43,51]. Immune reprogramming strategies such as bsAbs and ACT represent promising avenues to broaden therapeutic responsiveness in LCINS [73,76,81,82,83]. From a drug-development perspective, these observations underscore the need for immune strategies that do not rely on high baseline immunogenicity. Interpretation of LCINS studies should also consider the heterogeneity in the definition of never-smoker across studies, as smoking status is often self-reported and some datasets may include individuals with minimal or previous smoking exposure.

Finally, exploration of early detection and prevention frameworks integrating environmental, germline, and molecular risk factors may help reduce the growing global burden of LCINS [1,2,9]. Addressing these challenges will be essential to advance a unified model of precision oncology that acknowledges the biological uniqueness of LCINS and leverages both molecular targeting and immune modulation for durable disease control.

## 5 Conclusions

Experience from both clinical trials and translational studies indicates that LCINS cannot be approached in the same way as tobacco-related disease. Although molecularly targeted therapies form the backbone of treatment, long-term disease control is frequently undermined by acquired resistance and by progression within the CNS. Once kinase inhibitors fail, therapeutic options narrow substantially. In this setting, emerging modalities—including bsAbs, ADC, and adoptive cell–based approaches—offer new avenues to extend disease control in selected patients, particularly those with persistent oncogenic dependence. Continued integration of comprehensive molecular profiling with translational and clinical research will be essential to optimize sequencing, refine patient selection, and move toward more durable treatment paradigms in this biologically distinct population.

## Data Availability

Not applicable.

## Abbreviations

The following abbreviations are used in this manuscript:

ACT	Adoptive Cell Therapy
ADC	Antibody–Drug Conjugate
AEs	Adverse Events
ALK	Anaplastic Lymphoma Kinase
ALT	Alanine Aminotransferase
AST	Aspartate Aminotransferase
bsAbs	Bispecific Antibodies
CNS	Central Nervous System
CR	Complete Response
CTLA-4	Cytotoxic T-Lymphocyte-Associated Protein 4
DOR	Duration of Response
EGFR	Epidermal Growth Factor Receptor
ex20ins	Exon 20 insertion
FDA	Food and Drug Administration
HER2	Human Epidermal Growth Factor Receptor 2
HER3	Human Epidermal Growth Factor Receptor 3
HR	Hazard Ratio
ICIs	Immune Checkpoint Inhibitors
ILD	Interstitial Lung Disease
KRAS	Kirsten Rat Sarcoma Viral Oncogene Homologue
LAG–3	Lymphocyte-Activation Gene 3
LCINS	Lung Cancer in Never-Smokers
MEK	Mitogen-Activated Protein Kinase
MET	Mesenchymal-Epithelial Transition factor
METex14	Mesenchymal-Epithelial Transition Factor Exon 14 Skipping Mutation
M2	Macrophages
NSCLC	Non–Small Cell Lung Cancer
ORR	Objective Response Rates
OS	Overall Survival
PD-1	Programmed Cell Death Protein 1
PD-L1	Programmed Death-Ligand 1
PFS	Progression-Free Survival
QT	QT Interval
RET	Rearranged during Transfection
ROS1	c-Ros Oncogene 1
T cell	T Lymphocytes
TIGIT	T-cell Immunoreceptor with Immunoglobulin and Immunoreceptor Tyrosine-Based Inhibition Motif Domains
TILs	Tumor-Infiltrating Lymphocytes
TKIs	Tyrosine Kinase Inhibitors
TMB	Tumor Mutational Burden
TME	Tumor Microenvironment
TRK	Tyrosine Receptor Kinase
vs.	Versus
